# Climate change and timing of avian breeding and migration: evolutionary versus plastic changes

**DOI:** 10.1111/eva.12126

**Published:** 2013-11-12

**Authors:** Anne Charmantier, Phillip Gienapp

**Affiliations:** 1 Centre d'Ecologie Fonctionnelle et Evolutive, UMR 5175 Campus CNRSMontpellier Cedex 5, France; 2 Department of Animal Ecology, Netherlands Institute of Ecology (NIOO-KNAW)Wageningen, The Netherlands

**Keywords:** bird, climate change, evolution, phenology, phenotypic plasticity, selection, timing of breeding, timing of migration

## Abstract

There are multiple observations around the globe showing that in many avian species, both the timing of migration and breeding have advanced, due to warmer springs. Here, we review the literature to disentangle the actions of evolutionary changes in response to selection induced by climate change versus changes due to individual plasticity, that is, the capacity of an individual to adjust its phenology to environmental variables. Within the abundant literature on climate change effects on bird phenology, only a small fraction of studies are based on individual data, yet individual data are required to quantify the relative importance of plastic versus evolutionary responses. While plasticity seems common and often adaptive, no study so far has provided direct evidence for an evolutionary response of bird phenology to current climate change. This assessment leads us to notice the alarming lack of tests for microevolutionary changes in bird phenology in response to climate change, in contrast with the abundant claims on this issue. In short, at present we cannot draw reliable conclusions on the processes underlying the observed patterns of advanced phenology in birds. Rapid improvements in techniques for gathering and analysing individual data offer exciting possibilities that should encourage research activity to fill this knowledge gap.

## Introduction

Changes in the phenology of spring events are among the most frequently reported responses to climate change across all trophic levels and all types of freshwater, terrestrial and marine environments (e.g. Parmesan and Yohe 2003; Root et al. 2003; Thackeray et al. 2010). Thanks to the very long history of both scientific and public interest in ornithology, and the amenability of avian species to observation and capture, birds have contributed immensely to our knowledge on how animals are impacted by climate change. So far, this literature has been heavily focused on temperate species, while little is known on the response of tropical species. This strong bias will undoubtedly affect the conclusions we reach in this review. Most avian species and populations investigated have advanced their breeding and migration behaviours in the last decades (Møller et al. 2010; Knudsen et al. 2011). This common advancement in phenology is generally attributed to a response of timing of breeding and migration to changes in weather. Indeed, many bird species breed or migrate earlier in warmer springs (reviews in Gordo 2007; Lehikoinen and Sparks 2010). Timing of migration is also affected by other environmental variables such as favourable tailwinds (Alerstam 1990) or conditions at stop-over sites or wintering areas (Saino and Ambrosini 2008), which can be affected by climate change partly independently of temperature. However, in the context of studying climate change, the large majority of studies have discussed the observed changes with a focus on increased temperatures.

Phenological traits are generally expected to be closely related to individual fitness, especially in seasonal habitats such as temperate forests, where food availability is restricted to a short burst (Visser et al. 2006; Reed et al. 2013b). In these ecosystems, the seasonally changing environmental conditions set an ‘optimal time window’ for activities such as breeding, migrating or hibernating. This ‘optimal time window’ is determined by a variety of factors, of which the most relevant in the context of climate change are climatic factors and the phenology of other trophic levels (Visser and Both 2005). Many food resources show a clear seasonal trend in abundance or suitability. For example, the abundance of caterpillars, an important prey for many passerines during their reproductive period, shows a pronounced and short peak in spring in temperate European forests (Visser et al. 2006). Prey fish species of seabirds can also show distinct abundance peaks, for example, caused by spawning migration, thereby affecting the breeding success of zooplanktivorous and piscivorous seabirds (Sims et al. 2004; Hipfner 2008). Higher trophic level interactions can also affect optimal timing of important life history events. For example, the optimal time of migration in wader species depends partly on predation risk by birds of prey (e.g. Lank and Ydenberg 2003). Another possible factor affecting optimal timing is the timing of conspecifics, for example, through competition for breeding territories (e.g. Sergio et al. 2007) or social benefits of synchronized reproduction (Hatchwell 1991; Reed et al. 2006). Climatic factors can also directly affect the ‘optimal time window’ as cold spells in early spring can lead to mass mortality of early arriving migratory birds (Newton 2007). Consequently, climatic variables can induce selection on spring phenology of birds either indirectly by affecting processes at lower or higher trophic levels, or more directly by influencing their survival probability. Hence, in temperate zone organisms relying on strong seasonal changes in the ecosystem, one would expect climate change to alter selection on phenology and generally to lead to directional selection for earlier breeding and migration.

In fact, as far back as the late-1970s, correlative and experimental studies have repeatedly shown that reproductive success declines over the course of the breeding season in a variety of avian species, translating into strong directional selection for early breeding (review in Verhulst and Nilsson 2008). While a similar relationship is expected for migration time, not least because of the expected link between migration and breeding phenologies, far fewer studies have quantified selection on migration time due to the much higher effort required to record individual data on migration time as well as reproductive success and survival. The existing, but limited, evidence indicates that directional selection on timing of arrival at the breeding grounds favouring early arriving individuals is also common (Bêty et al. 2004; Smith and Moore 2005; Møller et al. 2010; Gienapp and Bregnballe 2012; Arnaud et al. 2013). Note however that modelling work suggests that selection need not always favour earlier migration when competition for territories is strong, even if the food peak advances (Johansson and Jonzen 2012).

Overall, there is strong evidence across a range of bird taxa for selection favouring early migration and breeding activity, and a general expectation of intensified selection in the face of climate change. In accordance with this selection for advanced phenology, there is overwhelming evidence across many bird taxa, for earlier mean migration dates and earlier mean breeding dates at the population level. The question addressed in this review is whether this evidence can be attributed to evolutionary changes in response to the documented selection forces, or whether it results from individual plasticity, that is, the capacity of individuals to adjust their phenology according to environmental cues informative about lower and/or higher trophic level phenology. First, we will briefly outline why it is important to distinguish between plastic and microevolutionary responses.

## Evolutionary versus plastic changes: why does it matter?

Phenotypic plasticity is defined as the ability of a genotype to produce different phenotypes in different environments (Pigliucci 2001, 2005). Many traits are phenotypically plastic and while ‘non-labile’ traits are stable during the life-time of an individual, for example, morphological traits which are influenced by conditions during ontogeny, ‘labile’ traits change repeatedly and can respond quickly to environmental conditions (e.g. behavioural or some life-history traits). Phenotypically plastic traits, and especially ‘labile’ traits, can hence respond very quickly to altered environmental conditions. This is especially obvious for phenology, the seasonal timing of life-cycle events (e.g. flowering, migration or breeding), where trait values (i.e. the date when these events occur) can vary by weeks from one year to the next.

Micro-evolutionary change as a response to selection acting on heritable traits is generally slower to change phenotypes than plasticity (Hendry and Kinnison 1999), in particular because heritabilities estimated in natural populations are often moderate (Kruuk 2004), and generation times can be on the order of several years. Consequently, phenotypic plasticity might be a better way to cope with environmental change as it can allow a faster tracking of the changing environment. As an illustrative example, a 47-year study of great tits *Parus major* breeding in nest-boxes in the United Kingdom has shown that over this half century, the mean egg-laying date of female great tits has advanced by about 14 days (Charmantier et al. 2008). This is equivalent to the advance in caterpillar phenology on the same study site during the same period, resulting in a close matching of bird and prey phenology. Based on estimates of laying date heritability, and on the force of selection presently acting on this trait, quantitative genetic models predict that selection would need to be four times stronger than the selection measured, for microevolution to explain the advance observed in great tit breeding phenology. Or in other words, the 14 day advance would take two centuries if it relied on an evolutionary adaptation alone. This example illustrates the main ‘advantage’ of individual plasticity compared to adaptive evolution to track climate change effects: plasticity ‘allows’ more rapid changes, as well as tracking of annual fluctuations in the environment.

**Figure 1:**
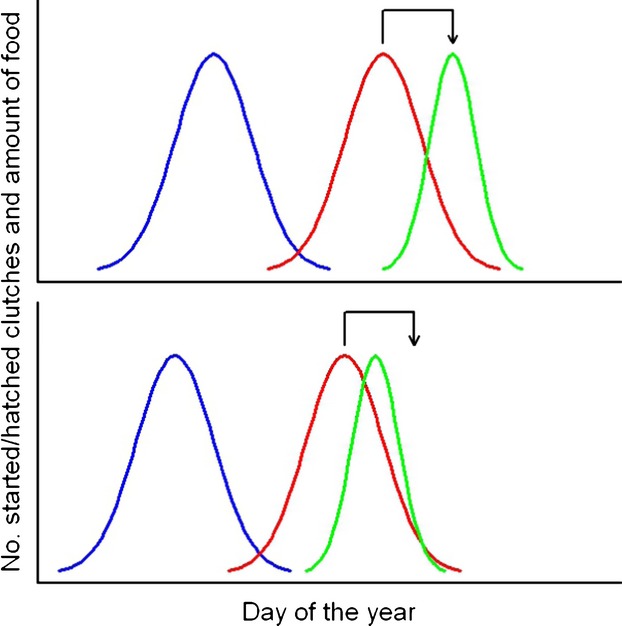
Schematic illustration of phenological ‘mismatch’ taking great tits and their caterpillar food supply as example. The curves indicate frequency distributions of first eggs of clutches (blue), hatching (red) and caterpillar abundance (green). Food demands of great tit chicks are highest approximately 9 days after hatching indicated by the black arrow. In the upper panel describing a scenario before climate change, the birds' breeding is well-timed to the caterpillars, and chick demand coincides with caterpillar abundance. In the lower panel, the timing of both the great tits and the caterpillars has advanced – due to climate change – but the caterpillars have advanced twice as fast. This now has led to a ‘mismatch’ between the chick demands and the phenology of the caterpillars.

However, for plasticity to remain efficient during an environmental change, it is required that the relationship between the environment that determines the trait (i.e. the ‘cue’), and the environment that determines fitness (i.e. the ‘selective environment’) remains the same during the selective process. When this is the case, the optimal reaction norm is not changed by environmental change. This however does not seem to be likely under continued climate change as the rate of climate change varies in space and time (Easterling 1997; Luterbacher et al. 2004) and individuals typically experience more than one season or region (e.g. in migratory species). The possible unreliability of cues guiding the plastic response is one of the major limits to the benefits of plasticity, although the exploration of costs and limits of phenotypic plasticity is very limited compared to investigations of benefits (DeWitt et al. 1998). For example, great tits react to temperatures in early spring to time their breeding, that is, they use these temperatures as ‘cues’. However, their reproductive success depends on the occurrence of caterpillars later in the season when the chicks' food demands are highest, and the phenology of these caterpillars is determined by temperatures in late spring (Fig. 1, Visser et al. 2006; Visser 2008). A differential change in these spring temperatures has occurred in the Netherlands, thereby altering the relationship between cue and optimum phenotype and leading to selection on the reaction norms (Nussey et al. 2005). Interestingly, even an equal rate of temperature increase in the two periods will lead to selection on the intercept of the birds' breeding time-reaction norm as the caterpillars respond more strongly to temperatures than the birds (T.E. Reed, P. Gienapp, and M.E. Visser, unpublished data).

## Are the changes adaptive in the face of climate change?

Adaptation is ultimately necessary for population persistence as sustained maladaptation can lead to extinction (Kopp and Hendry this issue; Gonzalez et al. 2013) unless counteracted by density dependence (Chevin and Lande 2010; Reed et al. 2013a). Phenotypic plasticity can allow a rapid adjustment to novel environmental conditions and even if this does not always allow a perfect tracking of the changing environment, as pointed out above, it could allow populations to persist in the short-term and ‘buy time’ for evolutionary adaptation in the long term (Chevin et al. 2010; Kovach-Orr and Fussmann 2013). Alternatively, plasticity can also hamper adaptation, in particular when new stressful environmental conditions trigger non-adaptive plasticity (Ghalambor et al. 2007). Hence, a better understanding of adaptive and non-adaptive phenotypic plasticity and genetic variation in natural populations is important to predict adaptation to climate change. As only adaptation will allow populations to persist, such predictions will allow a better assessment of possible threats of climate change to biodiversity. However, information about whether an observed response is due to plasticity or evolution will yield limited information towards this ultimate goal. Showing that a population is adjusting through phenotypic plasticity, for example, by demonstrating the absence of genetic change in the presence of phenotypic change, does not directly show that the observed phenotypic plasticity is ‘adaptive’, hence it does not guarantee that the population is ‘demographically safe’. The same applies for evolutionary responses: Evolutionary responses to environmental change may be too slow to rescue a population under sustained environmental change (Bürger and Lynch 1995; Chevin and Lande 2010; Gienapp et al. 2013). Besides disentangling phenotypically plastic and evolutionary responses to environmental change, we also need to know to what extent these responses are ‘fully adaptive’, that is, how much selection is acting on the trait under consideration.

In the particular case of responses to climate change, it is important to mention that many studies have documented selection favouring earlier breeding pairs before climate change was a recognized issue (Perrins 1965; Lack 1968; Price et al. 1988). Hence, demonstrating that earlier breeding is an adaptive response to climate change requires showing that selection for earlier breeding is stronger with (as opposed to without) climate change, in the absence of a less-than-perfect plastic response. This task involves a comparison of selection across years with differing climates or (preferably) identifying the factor(s) causing selection and showing that it is driven by climatic factors. The above cited studies on great tits provide such analyses. The study of Dutch great tits in Hoge Veluwe demonstrates that selection is driven by the temporal mismatch with the caterpillar food supply and that caterpillar phenology has advanced due to increasing spring temperatures (Fig. 1, Visser et al. 2006; Reed et al. 2013b). In the British tit study of Wytham woods, only warm springs show a strong directional selection for earlier breeding (see fig. 2A in Charmantier et al. 2008). Another example comes from pied flycatchers *Ficedula hypoleuca* where selection on breeding time is driven by spring temperatures during the arrival period of offspring and where these spring temperatures change due to climate change (M.E. Visser, P. Gienapp, A. Husby, M. Morrissey, I. de la Hera, F. Pulido, and C. Both, unpublished data). Studies demonstrating such statistical links between the strength of selection and the presence of climate change are unfortunately still scarce. The take-home message here is that we should be careful when inferring adaptive responses to climate change from the fact that selection and observed phenotypic change are in the same direction unless we are sure that selection is driven by a changing climate.

## Evolutionary versus plastic changes: which tools for avian phenology studies?

Disentangling evolutionary and phenotypically plastic changes can be done in a number of ways (see Merilä and Hendry this issue). Studying this in avian phenology poses special challenges but also offers certain advantages. In the following, we will scrutinize the approaches outlined by Merilä and Hendry (this issue, Table 1) for their applicability to study avian phenology in the context of adaptation to climate change.

**Table 1:** Empirical tests of evolutionary change or plastic responses underlying changes in avian timing of breeding or timing of migration

Species	Localization	Trait	Genetic change	Plastic change	Adaptive	Reference
Timing of breeding						
Collared flycatcher *Ficedula albicollis*	Gotland, Sweden	Laying date	No	Yes	Yes	Przybylo et al. (2000), Sheldon et al. (2003) and Brommer et al. (2005)
Pied flycatcher *Ficedula hypoleuca*	Hoge Veluwe, The Netherlands	Laying date	.	Yes	Yes	Both and Visser (2001, 2005)
Great tit *Parus major*	Hoge Veluwe, The Netherlands	Laying date	No	Yes	Yes	Nussey et al. (2005), Gienapp et al. (2006) and Husby et al. (2010)
	Wytham Woods, UK	Laying date	.	Yes	Yes	Charmantier et al. (2008) and Husby et al. (2010)
Blue tit *Cyanistes caeruleus*	D-Rouvière, France	Laying date	.	Yes	0	Porlier et al. (2012)
	D-Muro, France	Laying date	.	Yes	Yes	
	E-Muro, France	Laying date	.	Yes	0	
	E-Pirio, France	Laying date	.	Yes	Yes	
Guillemot *Uria aalge*	Isle of May, UK	Laying date	.	Yes	Yes	Reed et al. (2006)
	Southeast Farallon Island, USA	Laying date	.	Yes	Yes	Reed et al. (2009)
Song sparrow *Melospiza melodia*	Mandarte Island, Canada	Laying date	.	Yes	.	Wilson et al. (2007)
Common gulls *Larus canus*	Matsalu National Park, Estonia	Laying date	.	Yes	Yes	Brommer and Rattiste (2008) and Brommer et al. (2008)
Red-billed gull *Larus novaehollandiae scopulinus*	Kaikoura Peninsula, New Zealand	Laying date	No	.	Yes	Teplitsky et al. (2010)
Lesser kestrels *Falco naumanni*	Crau Plain, France	Settlement date	.	Yes	Yes	Mihoub et al. (2012)
Mauritius kestrel *Falco punctatus*	Mauritius	Laying date	.	Yes	Yes	Senapathi et al. (2011)
Timing of migration						
Barn swallow *Hirundo rustica*	Northern Italy	Spring arrival date	.	Yes	.	Saino et al. (2004)
	Badajoz, Spain	Spring arrival date	.	Yes	.	Balbontin et al. (2009)
Blackcap *Sylvia atricapilla*	Radolfzell, Germany	Timing of autumn migration activity	Yes (under artificial selection)	.	Yes	Berthold and Pulido (1994), Pulido and Berthold (2003, 2010)
American redstarts *Setophaga ruticilla*	Font Hill Nature Preserve, Jamaica	Spring departure date	.	Yes	.	Studds and Marra (2011)
Multiple	Powdermill Nature Reserve, USA	Spring arrival date	.	Yes	.	Van Buskirk et al. (2012)

Genetic change: this field can take the values ‘Yes’ (genetic change demonstrated), ‘No’ (demonstration of no genetic change) or ‘.’ (genetic change not tested). Plastic change: this field can take the values ‘Yes’ (individual plasticity demonstrated), ‘No’ (no plasticity demonstrated) and ‘.’ (plasticity not tested). Adaptive: this field can take the values ‘Yes’ (adaptive), ‘No’ (maladaptive), ‘0’ (neither adaptive nor maladaptive, for example, in the case there is no selection or selection is not significant) and ‘.’ (not investigated). We did not indicate the method of investigation separately by study in the table as all studies used the same methods: ‘animal models’ to test for genetic change, analysis of ‘individual plasticity in nature’ to test for plastic changes, and ‘phenotypic selection estimates’ to test whether changes were adaptive or not (see Merilä and Hendry this issue).

### Animal models

The potential for evolutionary change in a population facing a drastic environmental modification such as climate change is related to the amount of standing genetic variation for important adaptive traits, and also to the strength of selection acting on these traits. Indeed, in a quantitative genetics framework, two key models have been used to predict the evolution of selected traits. First, the so-called ‘breeder's equation’ predicts the response to selection (*R*) based on estimations of heritability (*h*²) and the strength of selection (*S*, Falconer and Mackay 1996): *R* = *h²S*. Ideally, this approach should be applied in a multivariate framework (the multivariate ‘Lande equation’), where the vector *R* of responses in a set of traits depends on the *G* matrix of additive genetic (co)variances for these traits and the selection gradients *β*: *R = G β* (Lande 1979). Second, the Robertson-Price Identity relates the evolutionary change (*R)* to the genetic covariance of the focal trait (*z*) with relative fitness (*w*): *R = σ*_*a*_*(z,w)* (Robertson 1966; Price 1970). Data on individual phenotypes, individual fitness, and relatedness between individuals in the focal population (i.e. a pedigree) are essential prerequisites for these estimations. Quantitative genetic analyses such as the animal model rely on phenotypic resemblance among relatives to estimate quantitative genetic parameters, such as heritability or genetic correlations (Falconer and Mackay 1996; Lynch and Walsh 1998). When studying wild bird populations, controlled breeding is usually impossible, hence quantitative genetic analyses rely on observing or reconstructing relatedness between phenotyped individuals. ‘Classical’ approaches are parent–offspring regression or full- or half-sib analyses where the degree of phenotypic similarity between offspring and parents or sibling groups is used to estimate additive genetic (co)variances. Animal models are better-suited for the study of natural populations as they can make use of the phenotypic information of relatives via the pedigree (Wilson et al. 2009).

In order to obtain individual data and assess heritability and selection, it is necessary to uniquely mark individuals, to follow them through their life-time (or at least a substantial part of it) and to reliably record the desired phenotypes, such as dates of initiation of breeding or dates of migration. Furthermore, it is also essential to link parents to their offspring to construct the pedigree. As birds can be easily individually marked by metal and coloured plastic leg-rings, and many avian species have extended brood care, in which often both parents take part, the necessary data for quantitative genetic analyses are comparably easily collected in birds. Several long-term studies have collected such data in some cases already for decades and they now offer a wealth of high-quality individual data suitable for sophisticated quantitative genetic analyses (Collins 2001; Postma and Charmantier 2007).

In theory, the best way to test for microevolution in real time is to estimate the individual genetic values, or breeding values, for the studied trait, and explore the changes in the mean breeding values over time (example in Garant et al. 2004; review in Kruuk 2004). In practice, however, the use of breeding values predicted from animal models can introduce serious biases in the analyses and conclusions reached regarding microevolution, because they do not always adequately reflect true breeding values. First, trends in predicted breeding values can mimic the observed phenotypic trend, even in the absence of a genetic change, if the phenotypic change over time is environmentally induced and this temporal variation is inadequately modelled (Postma 2006). Second, predicted values are always associated with (often large) standard errors, and they are non-independent for individuals of the same population, hence statistical care should be invested in controlling for this uncertainty and non-independence (Hadfield et al. 2010). Overall, the use of breeding values to test for microevolution requires special statistical attention and caution (see e.g. Milot et al. 2011).

### Individual plasticity in nature

‘Labile’ traits can be recorded several times in the same individual which allows the analyses of individual plasticity by modelling within-individual changes in response to the environment. Phenological traits, such as the seasonal timing of breeding or migration, are ‘labile’ traits when individuals breed or migrate in more than 1 year. Since recording individual data is comparably easy in birds, many studies analysed within-individual responses of avian breeding time (e.g. Nussey et al. 2005; Reed et al. 2006; Porlier et al. 2012) and migration time (Pulido 2007a,b; Knudsen et al. 2011), mainly in response to ambient temperature. A note of caution here is that although it seems straightforward to record timing of events, potential biases exist. For instance, many avian monitoring studies cannot identify a pair's identity before the parent's capture at the chick stage. Hence if the probability of failure in early stages of clutch initiation is high for early breeders, they will be observed as late rather than early breeders. The consequences of such biases on our estimation of plasticity, heritability and selection for phenological traits have never been explored.

The simplest form of a reaction norm is described by a linear regression of the response phenotype on the environmental variable known or thought to affect the analysed trait. Studies of individual plasticity commonly use this type of linear relationships implemented in mixed models (with individual identity as random effect). Individual variation in phenotypic reaction norms can be modelled by fitting the interaction between the environmental variable and the random effect individual identity (Henderson 1982; Pigliucci 2001; Nussey et al. 2007). These models allow for estimation of individual variation in each of the two parameters describing reaction norms, the elevation and the slope, as well as the covariance between them. While this approach is conceptually straightforward, there are some technical details which can require special attention. For example, it is necessary to centre the environmental variable individually, that is, that the mean environment for every individual has a value of zero, to reliably disentangle within- from among-individual changes (Kreft et al. 1995). Another important factor is the number of repeated records per individual as this affects the precision with which individual reaction norms will be estimated. Simulation models imply that generally large data sets with total number of observations in the hundreds are necessary, but also that the optimal power is attained with a number of individuals to observations per individual ratio around 0.5 (e.g. 10 individuals with 20 observations each for a total of 200 observations, Martin et al. 2011).

The ‘choice’ of the environmental variable against which phenotypes are regressed can also severely affect conclusions about variation in individual reaction norms. If an environmental variable is chosen that is only weakly correlated to the true causal factor for within-individual variation, the degree of phenotypic plasticity will be underestimated. Since temperature is the primary environmental cue for plant phenology (e.g. Bradshaw and Holzapfel 2006; Donnelly et al. 2012; Franks et al. this issue), it has been largely assumed that this should be also the case for the phenology of organisms at higher trophic levels, such as birds. However, it is now obvious that bird phenology depends on several cues that can be influenced by climate change (such as temperature) or not (such as photoperiod, Lambrechts et al. 1997) and that there is seldom an obvious measure of the climatic or weather conditions that causally affect a bird's phenological phenotype. In particular, although most birds breed earlier in warmer springs, there are a large number of possible measures of spring ‘warmness’. These will generally be correlated moderately or even (very) highly but which one is used as the environmental variable in a phenotypic plasticity analysis can strongly affect the results. For example, two studies analysed phenotypic plasticity in breeding time for the UK great tit population cited above, and depending on the period over which spring temperatures were averaged, individual variation in reaction norm slopes was present (Husby et al. 2010) or absent (Charmantier et al. 2008). Similarly, using very coarse-grained environmental variables which are probably only weakly correlated with the true causal factor will likely underestimate phenotypic plasticity and especially individual variation in reaction norms. This could be the case in Reed et al. (2006) study using the North-Atlantic-Oscillation Index, which summarizes climate variations across Western Europe and Eastern North America, in their analysis of breeding time in the common guillemot, *Uria aalge* showing no individual variation in reaction norm slopes. A similar problem is likely also present in the study of Van Buskirk et al. (2012) which analysed spring arrival timing of 27 North American bird species. Individual phenotypic plasticity of spring arrival in response to temperatures could only explain 13–25% of the advancement in phenology observed over 46 years. However, if the temperature measure was ‘too coarse’ and hence an inappropriate environmental variable, individual plasticity estimates could be downwardly biased. Identifying the correct environmental variable is especially difficult in migratory species as they often cover large distances in little time and the most relevant spatial and temporal scales across which environmental variables affect timing of migration is unclear.

When fine-grained environmental variables are used in analyses of individual phenotypic plasticity for avian phenology, they are commonly daily mean temperatures averaged over certain fixed calendar dates, which limits the possibility to extrapolate to other systems. These fixed periods are identical in all years for a given population, for example, from 15 February to 25 April in Husby et al. (2010) for the UK great tit population, which means that the predictive value of this proxy will co-vary with the absolute phenological timing in birds. This can be especially problematic when temperatures averaged over fixed periods are used to forecast phenology outside the current temperature range. A solution to this is to model the daily probability that an individual will start breeding depending on the environmental conditions up until that day using survival models that allow for so-called time-dependent variables, such as, for example, the ‘proportional hazards model’ (Cox 1972). This approach has so far been used to model phenotypic plasticity of timing of breeding, moult and migration in birds (Gienapp et al. 2005, 2010; Bauer et al. 2008; van de Pol and Cockburn 2011).

Another alternative to the use of average daily temperatures during fixed periods is the heat accumulation, that is, the temperature sums (in degree-days) above a threshold (e.g. 0°C) between a set start date (e.g. 1st January) and the phenological event of interest (e.g. Saino et al. 2011). If one were to apply this measure to an analysis of individual plasticity within populations, an interesting paradox may emerge where individual birds that are classically shown as being plastic in response to daily average temperatures would actually maintain the same relationship between their timing of breeding and the heat accumulation.

### Fine-grained population responses

Even if no individual data are available, a detailed analysis of phenotypic responses to the environment at the population level can contribute to disentangling plastic from genetic responses. While in most studies data on timing of breeding (e.g. date of egg-laying) can be linked to individuals because birds can often be caught at the nest, this information is often lacking in data on timing of migration because these data often consist more traditionally of counts of migrating birds at a given location. As pointed out by Merilä and Hendry (this issue) population mean reaction norms can be a first step to test for genetic change in a similar way to analyses of individual phenotypic plasticity. The rationale of this approach is that year-to-year fluctuations in mean population phenology are generally too fast to be interpreted as evolutionary responses to annually fluctuating selection pressures. As in an analysis of individual plasticity, there are some technical points to be taken into account. For example, it is of utmost important to de-trend both the observed phenotypes and the environmental variable prior to analysis (or to use formal time-series analyses). Otherwise, a correlation between mean phenology and the tested environmental variable can arise simply because both change in a similar fashion with time. If the observed advancement in phenology can be fully explained by a change in the environmental variable, plasticity becomes a strong candidate to explain the observed change, yet only an individual analysis as described above can conclude on the presence, strength and variation in individual plasticity.

The opposite case, that is, lack of correlation between annual environmental changes and annual changes in mean population, cannot be taken as unequivocal evidence for genetic change because there are several alternative explanations. First, as pointed out above, using an environmental variable that is only weakly correlated to the true causal factor as a covariate in phenotypic plasticity analyses will underestimate the degree of plasticity and give a downwardly biased slope of the reaction norm. This problem is especially prominent in studies of migration time as migrating individuals can quickly cover large geographical areas, which makes it difficult to identify a single meaningful environmental variable. Second, plastic phenotypes are likely to be affected by more than one factor. Avian breeding time, for example, is not only affected by weather but also by other intrinsic and extrinsic factors such as the individuals' age and condition (e.g. Komdeur 1996; Gonzalez-Solis et al. 2004; McCleery et al. 2008). Such factors that are either unidentified or cannot be included in the analysis might explain the decoupling between mean phenotypic change and measured environmental change. These issues will also apply to analyses of individual plasticity but due to the different statistical models used they are generally expected to be less problematic, not in the least because the higher quality data of individual-based studies allow including additional factors such as age.

### Other approaches

Merilä and Hendry (this issue) have listed other approaches that allow plasticity and microevolution to be disentangled as processes underlying the observed changes attributable to climate change, yet several of these are difficult if not impossible to implement when focusing on avian phenology. Laboratory studies on avian breeding time are possible and have been conducted in temperature- and/or photoperiod-controlled aviaries to identify environmental cues on the timing of egg-laying (Lambrechts et al. 1997; Visser 2008), as well as underlying physiological pathways involved (Visser et al. 2010). However, using temperature effects from such laboratory experiments to make predictions about phenotypically plastic responses to temperature in the wild is problematic due to the highly artificial conditions in the laboratory, such as *ad libitum* food or confinement stress. Studying true timing of migration in the laboratory is obviously impossible but proxies for this, for example migratory restlessness, have been used in artificial selection experiments in the blackcap *Sylvia atricapilla,* providing important insight in the potential for adaptive evolution in the timing of autumn migration (Pulido et al. 2001).

Common garden experiments are a powerful tool to test for genetic differentiation across space, and have, for example, been used in birds to test whether populations vary in their response to photoperiod (e.g. Berthold and Querner 1981; Silverin et al. 1993; Lambrechts et al. 1997). While common garden experiments are in theory equally well suited to test for genetic differentiation across time, by repeatedly testing the same population, this either requires long-term studies started a long time ago, or if started now, considerable long-term planning and effort. Consequently, contrarily to other taxa reviewed in this special issue (see in particular Collins et al., 2013) very few studies have used this approach in birds to test for genetic changes over time (but see an example in Pulido and Berthold 2010).

The most obvious and direct way to test for genetic change is to assess changes at the molecular genetic level. This however requires good knowledge of the molecular genetic architecture of the avian migration and breeding traits. Important progress has been made recently in understanding the molecular basis for the circadian clock regulating daily rhythms in invertebrates and vertebrates (including birds, see e.g. Johnsen et al. 2007). However, attempts to relate breeding phenology (Liedvogel and Sheldon 2010; Caprioli et al. 2012; Liedvogel et al. 2012) or migration phenology (Dor et al. 2011; Mueller et al. 2011) to *Clock gene* polymorphism or other candidate genes have so far yielded little. Undoubtedly, genomic approaches provide exciting opportunities to test for evolutionary responses of phenology to climate change, yet at present the lack of consistent relationships across species prevents any generalization. Note also that although identifying candidate genes may be the most direct route to documenting genetic change, it might not contribute to determining how much of the change in phenotype through time was the result of genetic change (Merilä & Hendry, this issue).

## Lessons from empirical studies of avian phenology

### Evolutionary potential of breeding and migration timing

Many long-term individual monitoring studies of birds have offered the possibility to estimate the heritability of timing of breeding, and it is now well known that egg-laying dates are commonly heritable [e.g. *h*² = 0.192 (SE = 0.036) in the collared flycatcher *Ficedula albicollis*, Sheldon et al. 2003; *h*² = 0.159 (SD = 0.059) in the great tit *Parus major*, McCleery et al. 2004; and *h*² = 0.145 in the common gull *Larus canus*, Brommer and Rattiste 2008] although two case studies have shown no significant heritability for female egg-laying dates (in the mute swan *Cygnus olor*, Charmantier et al. 2006; and in the red-billed gull *Larus novaehollandiae scopulinus*, Teplitsky et al. 2010). Similarly, a review of 12 avian studies (mostly laboratory based) showed that traits related to the timing of migration are commonly heritable, with a mean heritability of 0.34 (SD = 0.24) (Pulido 2007a,b). More strikingly, experiments inducing artificial directional selection on migratory activity in the blackcap *Sylvia atricapilla* induced a significant delay in the mean autumn departure date by the same order of magnitude than that predicted by the breeder's equation (Pulido et al. 2001), thereby confirming strong evolutionary potential for the timing of migration.

In summary, previous investigations in avian populations show that timing of breeding and timing of migration are heritable in many species, and in the face of climate change they are classically under strong negative directional selection. However, demonstrating that an avian phenological trait is heritable and is under directional selection only suggests that there is potential for adaptive evolution, but is insufficient to conclude that an advance of the average population laying date or arrival date from migration can be attributed to evolutionary adaptation (e.g. Teplitsky et al. 2010).

### A review of the literature

In order to assess the empirical evidence that supports individual plasticity versus microevolution as processes explaining changes in avian phenology (timing of breeding or timing of migration), we have reviewed the literature testing for one or both mechanisms. This review was based on an ISI Web of Science search using several combinations of key words for studies on breeding phenology (‘plasticity’+'phenology'+'bird', ‘evolution’+'phenology'+'bird', ‘evolution’+'laying date+'bird', ‘plasticity’+'laying date', ‘plasticity’+ ‘breeding time’+ ‘bird’, ‘evolution’ + ‘breeding time’+ ‘bird’ as of 01/04/2013, amounting to 863 records) and on migration phenology (‘evolution’ + ‘migration’ + ‘bird’, ‘plasticity’ + ‘migration’ + ‘bird’, 786 records). Table 1 offers an overview of studies where individual data have been used to test for plasticity and/or microevolution in response to a climate variable, and in a way that matches our descriptions above. In particular, we did not include in this table the many studies that claimed to show plasticity while using population trends rather than individual reaction norms. Results were interpreted by ourselves, and hence, there might be some discrepancies between statements in the cited references and our own interpretation, for example when adaptive plasticity is claimed but fitness has not been (adequately) measured. If data from the same population were analysed in different papers, thereby providing complementary results on plasticity, evolution and selection, they appear on the same table line and all the references are mentioned.

### Why so little evidence for an evolutionary response?

It is quite striking that compared to the very high number of hits in the ISI searches, studies of individual plasticity or microevolution of the timing of breeding and even more so the timing of migration, remain scarce (Table 1). There is however building evidence that birds from a range of taxa show a plastic advancement in their timing of migration and breeding, in response to warmer springs or summers. In the cases where selection analyses were conducted, they show that this advancement is overwhelmingly adaptive. In contrast, only three studies appear to have tested formally for an evolutionary genetic response to climate warming in the timing of breeding by exploring temporal trends in the breeding values of laying date, in a 23 year study of the collared flycatcher *Ficedula albicollis* (Brommer et al. 2005), in a 31 year study of the great tit *Parus major* (Gienapp et al. 2006) and in a 46 year study of the red-billed gull *Larus novaehollandiae scopulinus* (Teplitsky et al. 2010). These three studies have found no significant advancement of the mean predicted breeding values for the timing of reproduction. We do not know of an equivalent study in the timing of migration; however, a significant micro-evolutionary change in the timing of autumn migration activity has been measured in response to artificial selection in captive blackcaps *Sylvia atricapilla* (Pulido and Berthold 2003, 2010), along with an even stronger reduction in migratory activity. We could add here that several other papers, although not formally testing changes in breeding values, have shown that phenotypic changes in phenology could be explained entirely by individual plasticity (Charmantier et al. 2008). Hence, the evidence we have reviewed here points to the conclusion previously reached (Gienapp et al. 2007, 2008; Sheldon 2010) that we do not have any good evidence pointing to contemporary climate-change evolution in bird phenology, while there is ample evidence for strong individual plasticity in avian phenology (Table 1). However, it would be premature to interpret this lack of evidence as revealing that microevolutionary responses to climate change are rare; our assessment instead points towards a paucity of investigations, limiting the scope for generalizations to be made at this stage.

A detailed look at the references from the ISI searches shows that only 10% of studies on avian migration time that reported an advancement of timing were based on individual data (seven of 66, see Supporting information, Table S1). This leads to the worrying conclusion that many studies claiming to support climate-driven evolutionary change do not even measure individual plasticity (e.g. Jonzen et al. 2006). The general deficiency of appropriate tests suggests that either longitudinal individual data allowing proper investigations are lacking, or that they have not been properly analysed. It is obvious that annual average phenological dates are easier to collect than individual data. However, Møller and Fiedler (2010) have estimated that there exist more than 200 long-term (>10 years) time series based on individually marked birds (most of them providing pedigrees), and maybe ten times this if data collected by amateurs were shared for data analysis. A great majority of these time series will include data on avian phenology. Hence, collective sharing of data in collaborative studies including experts in the analyses of microevolution, plasticity and selection could well soon trigger a multitude of new results and bring this field to a whole new scale.

## Conclusions and future directions

As outlined in the previous section, there is an overall critical lack of investigations on microevolutionary changes in bird phenology in response to climate change, similarly to what is found in other vertebrates (see Boutin and Lane this issue; Urban et al. this issue and Crozier and Hutchings this issue, for mammals, amphibians/reptiles and fish, respectively). However, in the case of birds, many studies cited in Table 1 are based on available phenotypic and pedigree data that would allow such scrutiny. Hence, our first encouragement will be to get back to these data sets and test for evolutionary changes, following the latest statistical recommendations (Postma 2006; Hadfield et al. 2010). We briefly list below some other exciting perspectives that we would prioritize to improve our knowledge on the underlying processes explaining the general advancement in observed bird phenology.

### Maintaining high priority on obtaining more individual data

While egg-laying dates are already commonly part of long-term monitoring programmes, a better understanding of evolutionary processes shaping variation in migration behaviour requires similar individual data (see Dittmann and Becker 2003 for an example of exact individual arrival dates in a long-term project). The rapid improvement and accessibility of satellite-tracking and geolocator devices (Gillespie 2001; Wakefield et al. 2009) will soon allow researchers to record entire migration journeys in larger samples of birds, thus opening stimulating new possibilities on the study of individual variation in the various behaviours involved in the migration route and timing.

### Developing multi-trait measures and analyses

While this review focused on the timing of migration and breeding, climate change affects other important traits in birds, such as migration activity (Pulido and Berthold 2010), the probability of laying multiple broods (Husby et al. 2009), intermittent breeding or the propensity to skip breeding altogether, (Cubaynes et al. 2011), body size (Teplitsky and Millien this issue) and survival (Grosbois et al. 2006). Since many of these traits interact at a phenotypic and possibly genetic level with avian phenology, for example, via life-history trade-offs, they should be involved as much as possible in both the data collection and analytical process.

### Exploring evolution of plasticity

Climate change not only results in increased temperatures but also in more frequent extreme climatic events (Tebaldi et al. 2007). Hence, it is highly probable that birds are selected not only for earlier phenology but also for a fine-tuned adjustment in their phenology every year. In fact, developing a reaction norm perspective when investigating microevolutionary response to climate change might reveal that adaptation results mainly from a heritable change in individual reaction norms rather than in the mean phenotypic response. Several studies in Table 1 have already attempted to measure the variation in reaction norm properties across individuals (*G* × *E* interactions when reaction norm show heritable variation), and selection acting on these properties (Brommer et al. 2005; Nussey et al. 2005; Reed et al. 2006; Wilson et al. 2007). Although considering variation in phenological plasticity adds computational complexity and requires large datasets with repeated individual data, it will most probably be an essential contribution to our understanding of how bird populations can adapt to the increasing weather variability.

### Exploring responses across populations of the same species

Due to their time-consuming nature, avian individual monitoring projects are often based on single study sites. However, the rare projects with comparisons across populations allow researchers to assess how generalizable single-population plasticity trends are (Porlier et al. 2012) but also to understand which factors drive differences in plasticity and evolution across a species range (Both et al. 2006). Since avian demographic responses to climate change are not uniform across species ranges (Jiguet et al. 2010) future studies should develop comparisons across the species thermal range. The study of populations at range-edge location may be of particular conservation value because they represent significant components of intraspecific biodiversity (Hardie and Hutchings 2010) and potential sources of evolutionary innovation and persistence during rapid environmental change such as global warming (Sexton et al. 2009).

### Diversifying the ecological niches explored

Table 1 shows a strong bias towards temperate insectivorous birds, while birds in many other ecosystems are completely unexplored because of lack of individual data. In particular, we know that some extreme areas such as the Arctic are showing strong signs of climate warming (Moritz et al. 2002) and previous studies have shown that Arctic birds can modulate the sensitivity of their hormonal response to local environmental conditions in order to fine-tune the onset of their very brief territoriality (Wingfield and Hunt 2002). This provides grounds to predict that Arctic birds may display particularly strong plasticity.

### Linking phenological changes to population demography

Besides our main goal of improving our knowledge as evolutionary biologists on population adaptive responses to climate change, we are increasingly under the requirement from decision makers to forecast future changes and to offer advice on conservation strategies. Understanding the respective roles of individual plasticity and evolutionary responses in the phenological changes observed is an initial crucial step in order to offer predictive scenarios (Jenouvrier 2013), since as we have discussed, the two mechanisms act at different time-scales. However, a further essential step is to gauge the demographic consequences of adaptation or maladaptation by bridging the gap between evolutionary ecology and demography. Although this eco-evolutionary dynamics approach is at its infancy (see also recommendations in Reusch this issue), it should soon promote the possibility to predict possible effects of climate change on population dynamics, such as two recent studies modelling the contribution of phenotypic plasticity (Vedder et al. 2013) and phenological mismatch (Reed et al. 2013a), to population growth and persistence.

## Supporting Information

**Table S1.** Studies reporting significant advancements in timing of avian migration.
