# Supplementary material for: Climate change and timing of avian breeding and migration: evolutionary versus plastic changes
Source: Evol Appl. 2013 Nov 12;7(1):15–28. doi: 10.1111/eva.12126 (PMC3894895; doi:10.1111/eva.12126)
Supplement: Supplementary file 1 [file eva0007-0015-SD1.docx]

**Table S1.** Studies reporting significant advancements in timing of avian migration. Relevant results from ISI Web of Science search using the following search terms: migration AND (time OR timing) AND bird AND "climate change" (266 hits). We specify whether individual data is included in the analysis (Ind. data : N for ‘no’, Y for ‘yes’) and give a short description of the main results.

| Species | Trait(s) | Ind. data | Short description | Reference |
| --- | --- | --- | --- | --- |
| waders | - spring & autumn passage date | N | - spring migration generally advanced, less clearer pattern in autumn migration  - correlation of spring & autumn migration with NAO | Adamik & Pietruszkova (2008) Acta Ornithol. 43, 119-128 |
| barn swallow | - arrival at and departure from wintering area | N | - departure date advanced | Altwegg et al. 2012 Proc. R. Soc. B 279, 1485-1490 |
| *Tringa* sandpipers | - passage date | N | - advancement in spring and delay in autumn  - correlation with weather variables and demographic parameters | Anthes 2004 Bird Study 51, 203-211 |
| *Sylvia* warblers | - first arrival date | N | - advancement & correlation with weather variables but unclear whether these can explain advancement | Askeyev et al. 2009 Central Eur. J. Biol. 4, 595-602 |
| curlew sandpiper | - autumn passage date | N | - migration time depends on summer temp, breeding success (and predation)  - advanced by 0.6 days per year | Barshep et al. 2012 Ornis Fenn. 89, 120-129 |
| multiple | - first arrival date  - last departure date | N | - changes in timing  - correlation with weather variables not analysed | Beaumont et al. 2006 Global Change Biol. 12, 1339-1354 |
| pied flycatcher | - timing of migration | N | - recovery date in N-Africa advanced (indicating earlier departure) but arrival at breeding grounds has changed little | Both (2010) Curr. Biol. 20, 243-248 |
| bar-tailed godwit | - departure date from wintering area | Y | - departure date correlated with latitude of breeding area | Conklin et al. 2010 Nature Comm. 1, 67 |
| bar-tailed godwit | - migration schedule | Y | - analysis of individual repeatability of migratory schedule | Conklin et al. 2013 PLoS 1 8, e54535 |
| multiple | - passage | N | - advancement of spring and autumn migration & correlation with weather variables | Cotton 2003 PNAS 100, 12219-12222 |
| multiple | - first arrival dates | N | - general advancement & correlation with weather variables  - not tested whether weather variables can explain trend | Croxton et al. (2006) Acta Ornithol. 41, 103-111 |
| multiple | - spring arrival date | N | - advancement & correlation with weather variables | DeLeon et al. 2011 Condor 113, 915-923 |
| house martin | - first arrival dates | N | - advancement & correlation with spring temperature  - not entirely clear whether increasing spring temp. can explain advancement | Dolenec & Dolenec 2011 Zoologia 28, 139-141 |
| blackcap | - first arrival dates | N | - advancement & correlation with spring temperature  - not clear whether increasing spring temp. can explain advancement | Dolenec & Dolenec 2010 Polish J. Zool. 58, 605-608 |
| multiple | - spring arrival date | N | - general advancement & correlation with temperature in long-term data set (157 years) | Ellwood et al. 2010 Condor 112, 754-762 |
| common crane | - autumn passage date | N | - migration advanced and was related to climate variables | Filippi-Codaccioni et al. 2011 Acta Ornithol. 46, 37-45 |
| multiple | - spring arrival | N | - correlation with NAO | Forchhammer et al. 2002 J. Anim. Ecol. 71, 1002-1014 |
| multiple | - arrival and departure date | N | - winter residents arrived later & departed earlier, less clear trends in summer residents | Foster et al. (2010) Wilson J. Ornithol. 122, 116-112 |
| white-fronted goose | - departure date from wintering areas | N | - advancement of departure date uncorrelated to temperature at wintering area(s)  - no warming at staging area (Iceland)  - improvement of body condition at departure from wintering area(s) despite no warming | Fox & Walsh 2012 Hydrobiol. 697, 95-102 |
| multiple | - spring & autumn migration (sightings) | N | - arrival advanced & correlated with weather variables – explanation of trends not tested  - less clear pattern for autumn migration | Gordo & Sanz 2006 Global Change Biol. 12, 1993-2004 |
| multiple | - spring arrival date | N | - advancement & correlation with African weather variables | Gordo et al. 2005 Global Change Biol. 11, 12-21 |
| multiple | - first arrival date | N | - advancement & correlation with local spring temperature and winter (NAO?) | Gunnarsson & Tomasson 2011 Bird Study 58, 1-12 |
| multiple | - spring passage date | N | - general advancement & correlation with NAO  - not tested whether NAO explains advancement | Hüppop & Hüppop 2003 Proc. R. Soc. B 270, 233-240 |
| multiple | - autumn passage date | N | - advancement in Sub-Saharan migrants, delay in other species | Jenny & Kery 2003 Proc. R. Soc. B 270, 1467-1471 |
| multiple | - spring arrival date | N | - long-distance migrants have advanced their spring arrival more than short-distance migrants | Jonzén et al. 2006 Science 312, 1959-1961 |
| multiple | - arrival and departure from wintering area | N | - later arrival and earlier departure  - data from "citizen science" | Kobori et al. 2012 Ecol. Res. 27, 173-180 |
| blackcap, garden warbler | - passage date | N | - spring migration advanced in garden warblers but not in blackcaps  - juvenile garden warblers delayed autumn migration | Kovacs et al. 2011 J. Ornithol. 152, 365-373 |
| nightingale | - first arrival date | N | - advancement | Kralj & Dolenec (2008) Cent. Eur. J. Biol. 3, 295-298 |
| multiple | - arrival date | N | - advancement | Ledneva et al. Wilson Bull. 116, 224-231 |
| waterfowl | - autumn passage date | N | - migration time delayed in most species but advanced in bean goose | Lehikoinen & Jaatinen 2012 J. Ornithol. 153, 563-570 |
| sparrowhawk | - spring & autumn migration  - breeding time | N | - advancement of spring & autumn migration and breeding time  - no change in migration distance | Lehikoinen et al. 2010 J. Avian Biol. 41, 627-636 |
| black-tailed godwit | - timing of staging  - arrival date  - egg-laying date | Y | - individual timing repeatable  - timing of staging, arrival date & laying date not correlated | Lourenco et al. 2011 J. Ornithol. 152, 1023-1032 |
| multiple | - spring arrival | N | - general advancement & correlation with weather variables | Macmynowski (2007) Global Change Biol. 13, 2239-2251 |
| multiple | - spring passage date | N | - moderate advancements | Marra et al. 2005 Oecologia 142, 307-315 |
| multiple | - autumn passage date | N | - long-distance migrants advanced, no change in short-distance migrants | Mezquida et al. (2007) Ardeola 54, 251-259 |
| multiple | - spring passage date | N | - advancement & correlation with August temperature | Miholcsa et al. (2009) Acta Zool. Acad. Scie. Hung. 55, 175-185 |
| multiple | - passage date | N | - few advancements in spring passage, more delays in autumn | Mills 2005 Ibis 147, 259-269 |
| barn swallow | - first arrival & last departure at wintering area | N | - arrival advanced  - "tendency to delay in last departure" | Møller et al. 2011 Clim. Res. 47, 201-205 |
| multiple | - spring arrival date | N | - general advancement & correlation with weather variables  - not tested whether weather variables explain advancement | Murphy-Klassen et al. (2005) Auk 122, 1130-1148 |
| Eider | - arrival date  - migration strategy | Y | - individuals followed one of three distinct migration strategies | Petersen 2009 Condor 111, 59-70 |
| multiple | - spring passage date | N | - timing advanced and was related to NDVI (negatively: later in good years)  - not tested whether advancement can be explained by NDVI | Robson & Barriocanal 2011 J. Anim. Ecol. 80, 320-331 |
| Kirtland's warbler | - arrival date  - breeding time | Y | - arrival and reproductive success depends on climate in wintering area  - no time trends in timing reported | Rockwell et al. 2012 Auk 129, 744-752 |
| sharp-shinned hawk | - autumn passage date | N | - delayed autumn migration | Rosenfield et al. 2011 Wilson J. Ornithol. 123, 154-158 |
| multiple | - spring migration | N | - general advancement | Rubolini et al. 2007 Clim. Res. 35, 135-146 |
| multiple | - first arrival dates | N | - some advancements & correlations with weather variables  - not tested whether weather variables explains trends | Rubolini et al. (2007) Int. J. Biometeorol. 51, 553-563 |
| multiple | - spring passage date | N | - advancement & correlation with weather variables  - weather variables could not fully explain advancement | Saino et al. 2007 Clim. Res. 35, 123-134 |
| Hudsonian godwit | - arrival date at breeding area | N | - arrival date advanced in one and delayed in another population  - cues for one advancing population remained reliable  - cues for delaying population became unreliable | Senner 2012 Auk 129, 670-682 |
| multiple | - spring and autumn passage date | N | - spring migration generally advanced  - less changes in autumn migration  - relationship with weather variables | Smith & Smith 2012 Emu 112, 333-342 |
| multiple | - autumn passage date | N | - autumn migration generally delayed | Smith & Paton 2011 Wilson J. Ornithol. 123, 557-566 |
| multiple | - passage date | N | - spring migration advanced & correlated to weather variables  - unclear whether weather variables can explain advancement  - no changes in autumn migration | Sokolov 2006 Zool. Zhurnal 85, 317-341 |
| wood thrush | - departure date from wintering area  - arrival date at breeding area | Y | - departure date strongly determines arrival date  - spring migration timing repeatable | Stanley et al. 2012 PLoS 1 7, e40688 |
| multiple | - spring passage date | N | - general advancement & correlation with NAO  - not tested whether advancement can be explained by NAO | Stervander et al. 2005 J. Avian Biol. 36, 210-221 |
| Whooper swan | - departure from wintering area | N | - departure date advanced in response to warmer temperatures (& population size) | Stirneman et al. 2012 Ibis 154, 542-553 |
| multiple | - arrival time | N | - some advancements | Strode 2003 Global Change Biol. 9, 1137-1144 |
| American redstart | - departure from wintering area | Y | - departure date depends on weather variables (rainfall) in wintering area | Studds & Marra 2012 Proc. R. Soc. B 278, 3437-3443 |
| multiple | - first arrival dates | N | - general advancement | Swanson & Palmer 2009 J. Field Ornithol. 80, 351-363 |
| sedge, reed and marsh warbler | - spring and autumn passage date | N | - spring migration advanced  - autumn migration delayed | Szilvia et al. 2012 Central Eur. J. Biol. 7, 115-125 |
| multiple | - passage dates | N | - general advancement | Thorup et al. (2007) Oecologia 151, 697-703 |
| pink-footed goose | - departure from wintering / staging sites | (Y) | - departure date from DK advanced  - correlation with 'spring' NDVI  - 'spring' advanced at several areas and was correlated among areas  not really a formal analysis of plasticity but indicative | Tombre et al. 2008 J. Avian Biol. 39, 691-703 |
| barnacle goose | - departure from wintering / staging sites | N | - departure from wintering area advanced(?)  - not correlated with 'spring'  - 'spring' not correlated among areas | Tombre et al. 2008 J. Avian Biol. 39, 691-703 |
| multiple | - autumn passage date | N | - no general change | Tottrup et al. 2006 Ardea 94 (S1) 527-536 |
| multiple | - spring passage date | N | - general advancement | Tottrup et al. 2006 J. Avian Biol. 37, 84-92 |
| multiple | - passage date at ringing station | N | - passage date was related to temperature & advanced  - temperature changes can't fully explain advancement | van Buskirk et al. 2012 Ecol. Evol. 2, 2430-2437 |
| blackcap | - arrival date (and others) | N | - arrival advanced | Wesolowski 2011 J. Ornithol. 152, 319-329 |
| multiple | - first arrival date | N | - analysis of correlation with weather variables | Zalakevicius et al. 2006 J. Ornithol. 147, 326-343 |
| steppe eagle | - spring passage date | N | - advancement & correlation with weather variables | Zduniak et al. (2010) Clim. Res. 42, 217-222 |
